# Target-driven DNA association to initiate cyclic assembly of hairpins for biosensing and logic gate operation†
†Electronic supplementary information (ESI) available: Supplementary table and figures. See DOI: 10.1039/c5sc01215e


**DOI:** 10.1039/c5sc01215e

**Published:** 2015-05-12

**Authors:** Yuehua Guo, Jie Wu, Huangxian Ju

**Affiliations:** a State Key Laboratory of Analytical Chemistry for Life Science , School of Chemistry and Chemical Engineering , Nanjing University , Nanjing 210093 , P. R. China . Email: hxju@nju.edu.cn ; Fax: +86 25 83593593 ; Tel: +86 25 83593593

## Abstract

A target-driven DNA association was designed to initiate cyclic assembly of hairpins, which led to an enzyme-free amplification strategy for detection of a nucleic acid or aptamer substrate and flexible construction of logic gates. The cyclic system contained two ssDNA (S1 and S2) and two hairpins (H1 and H2). These ssDNA could co-recognize the target to produce an S1–target–S2 structure, which brought their toehold and branch-migration domains into close proximity to initiate the cyclic assembly of hairpins. The assembly product further induced the dissociation of a double-stranded probe DNA (Q:F) *via* toehold-mediated strand displacement to switch the fluorescence signal. This method could detect DNA and ATP as model analytes down to 21.6 pM and 38 nM, respectively. By designing different DNA input strands, the “AND”, “INHIBIT” and “NAND” logic gates could be activated to achieve the output signal. The proposed biosensing and logic gate operation platform showed potential applications in disease diagnosis.

## Abstract 2

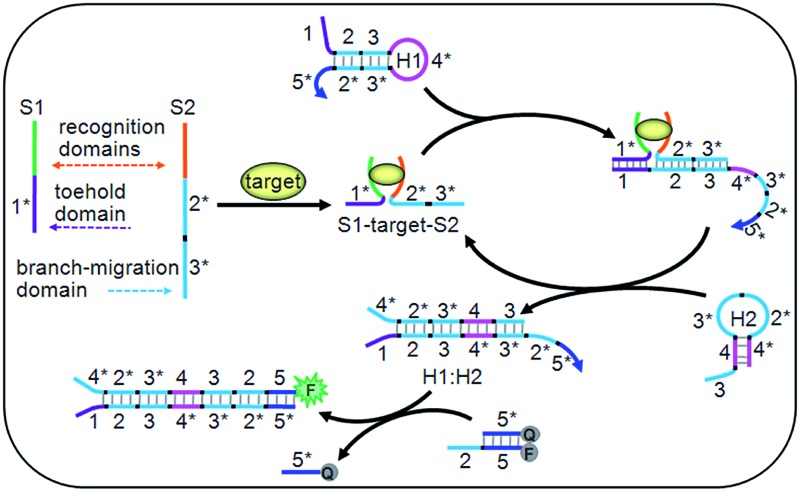
Target-driven DNA association is designed for initiating the cyclic assembly of hairpins for target detection and logic gate operation.

## Introduction

Due to its exquisite recognition properties relying on Watson–Crick base-pairing, DNA can be programmed and assembled in a predictable manner.^1^ Along with the capabilities of outstanding data storage and flexibility in design, DNA has been considered as one of the most promising building blocks in nanotechnology.^2,3^ Numerous machine-like DNA devices, for example nucleic acid tweezers,^4,5^ walkers,^6,7^ logic gates and circuits,^8–10^ catalytic amplifiers,^11,12^ and sensors,^13,14^ have been programmed. These DNA machines can be activated by different inputs, such as fuel DNA strands,^15^ H^+^/OH^–^,^16^ and metal ions,^17^ and have been applied for controllable drug delivery,^18,19^ detection of targets *in vitro* and *in vivo*,^20^ and programmed synthesis.^21^

Most of the DNA machines are programmed with a toehold-mediated DNA strand displacement strategy,^1,7,10–12,19^ in which a short single strand region at one end of a double helix strand called the toehold domain binds to its complementary strand and juxtaposes a longer segment of single-stranded DNA called the branch-migration domain close to the duplex to displace the strand with the same sequence as the branch-migration domain.^22,23^ Combining with different ribozymes, autocatalytic or cross-catalytic DNA circuits have been constructed to achieve exponential signal amplification in biorecognition process.^24–26^ However, these enzyme-based amplification schemes normally require harsh experimental conditions, complicated instrumentation and even complex thermal-cycling procedures.

**Fig. 1:**
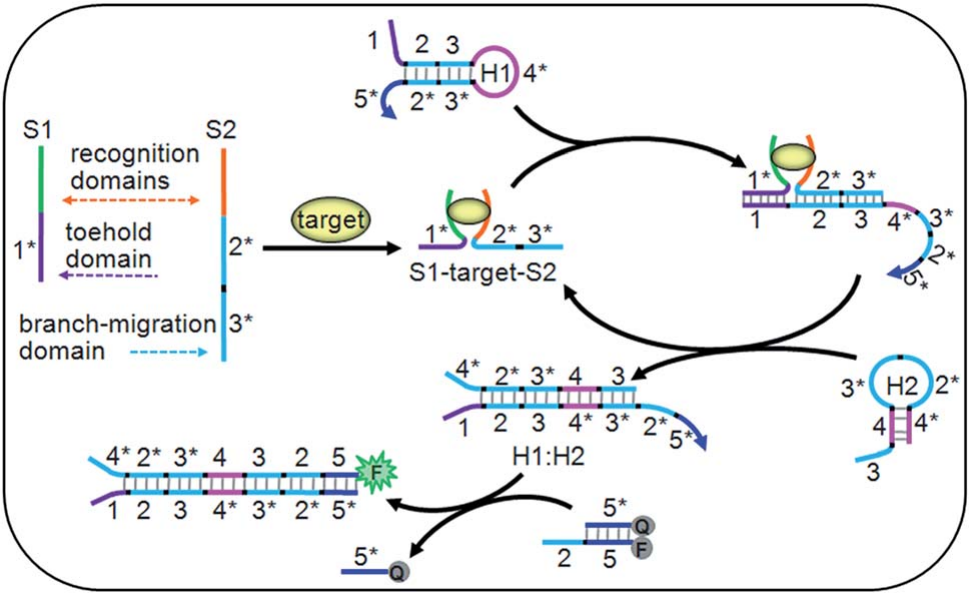
Schematic diagram of the target-driven DNA association to cyclically mediate the assembly of hairpins.

Recently, the advances in molecular programming have yielded non-enzymatic DNA circuits for amplifying and transducing signals,^27–29^ which is appealing for the development of low-cost point-of-care diagnostics. For example, the catalyzed hairpin assembly (CHA), in which two pre-designed hairpins that are without interactions can be catalyzed to form a duplex *via* toehold-mediated strand displacement, has been engineered to yield hundreds-fold amplified signals with negligible background.^30–34^ This work designed a target-driven DNA association to initiate the enzyme-free cyclic hairpin assembly, which could greatly amplify the signal for sensitive detection of the target, such as DNA or an aptamer substrate, adenosine triphosphate (ATP) (Fig. 1). Owing to the presence of different input strands in the toehold-mediated strand displacement,^35–38^ which has been applied to the design of a four-way DNA junction-driven toehold-mediated strand displacement for building a DNA logic circuit,^37^ this work further designed “AND”, “INHIBIT” and “NAND” logic gates, which could be activated using the target DNA-driven association, thus flexible and sensitive operations of signal input and output were achieved.

The DNA association initiated cyclic system contained two ssDNA (S1 and S2), two hairpins (H1 and H2) and a double-stranded probe DNA (Q:F) (Table S1 in ESI†). Particularly, the toehold and branch-migration domains lie on S1 and S2, and were connected to the recognition domains respectively. S1 and S2 could co-recognize the target to produce an S1–target–S2 structure, which brought the toehold (1*) and branch-migration (2*3*) domains into close proximity to hybridize with the 123 region of H1. The 3*4* region of the opened H1 further hybridized with the 34 region of H2 to open H2, and the hybridization of the 2*3* region of H2 with the 23 region of H1 led to the release of the S1–target–S2 structure, which initiated the cyclic hairpin assembly. The 2*5* region of H1 in the assembled product could switch the fluorescence signal for target detection. Due to the cyclic amplification, the cyclic system could detect the DNA target down to pM levels and ATP down to nM levels, which is comparable to those with complex enzymatic amplifications.^39–41^ By designing the recognition regions of S1 and S2, the proposed system could be conveniently extended to other targets, such as proteins, RNA and metal ions, showing potential applications.

## Results and discussion

### DNA detection

**Fig. 2:**
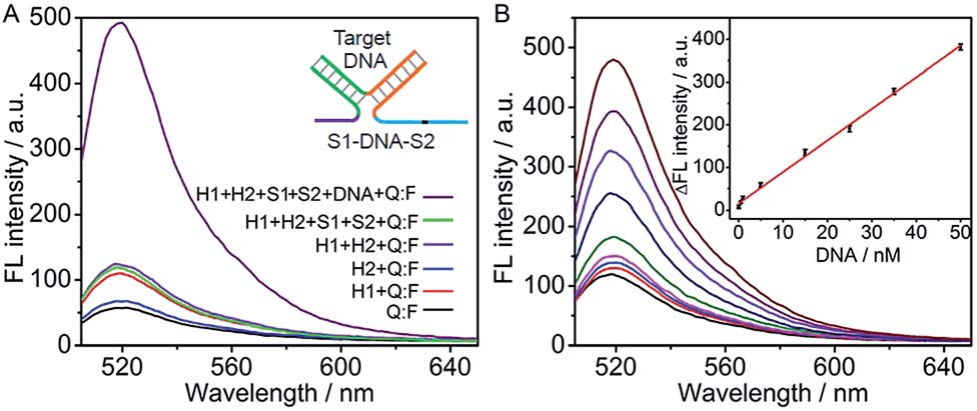
(A) Fluorescence spectra of different solutions containing the marked components at 200 nM H1 and H2, 100 nM S1 and S2, 200 nM Q:F and 50 nM target DNA. (B) Fluorescence spectra at 0, 0.05, 0.5, 1, 5, 15, 25, 35, 50 nM target DNA (from bottom to top). Inset: calibration curve for DNA detection.

The DNA association initiated cyclic system was firstly used for detection of target DNA. The recognition domains of S1 and S2 were designed to have 16 and 17 complementary bases to two ends of the target DNA, respectively. Their hybridization with target DNA led to the association of the toehold and branch-migration domains in the S1–DNA–S2 complex (inset, Fig. 2A), which initiated the hairpin assembly and produced a fluorescence signal. The formation of the three-strand complex was verified by polyacrylamide gel electrophoresis (PAGE) experiment (Fig. S1 in ESI†). In the absence of target DNA, S1, S2 and H2 hardly affected the fluorescence spectrum of Q:F (the green and the blue lines) (Fig. 2A). The slight fluorescence increase in the sample of “H1 + Q:F” (the red line) was likely caused by a small fraction of mis-formed (mis-synthesized and/or mis-folded) H1 that interacted with the probe Q:F duplex directly to separate the fluorophore- and quencher-bearing strands.^32^ In the presence of target DNA the fluorescence spectrum showed a high intensity, which confirmed the feasibility of the cyclic system for detection of target DNA.

The DNA assay conditions including the concentrations of Mg^2+^, hairpins and Q:F duplex and reaction time were optimized (Fig. S2 in ESI†). Here, Mg^2+^ is added in the buffer to improve the DNA hybridization. However, at high concentration of Mg^2+^, hairpin H2 could open a large amount of hairpin H1, resulting in a high background signal.^31,42^ The ratio of signal to background (*F*/*F*_0_) increased with the increasing concentrations of Mg^2+^, hairpins and Q:F duplex, and then tended to the maximum value at 6 mM Mg^2+^, 200 nM H1 and H2, and 200 nM Q:F, respectively. Further increase in their concentrations led to a decrease of the *F*/*F*_0_ due to the enhanced background. Hence, these concentrations were used in the detection of DNA as well as the design of DNA logic gates in the following section. With these optimal conditions, the reaction time of 60 min was also selected.

Under the optimized conditions, the fluorescent intensity of the cyclic system increased upon the addition of different concentrations of DNA target (Fig. 2B). The absolute fluorescence intensity (Δ*F* = *F* – *F*_0_, *F*_0_ and *F* are the fluorescence intensities detected in the absence and presence of target DNA respectively) was proportional to the target DNA concentration ranging from 0.05 to 50 nM (*R* ^2^ = 0.9959) (inset, Fig. 2B). The detection limit estimated at 3*σ* was calculated to be 21.6 pM, which was 5 times lower than that using nicking and polymerase enzymes to generate autonomous replication/nicking machinery for DNA detection^39^ and similar to that of the previous fluorescence method using enzymatic amplification.^40,41^ Meanwhile, the proposed method is time-saving in comparison with the reported amplified detection of DNA through the enzyme-free autonomous assembly by DNAzyme wires.^43,44^

### ATP detection

**Fig. 3:**
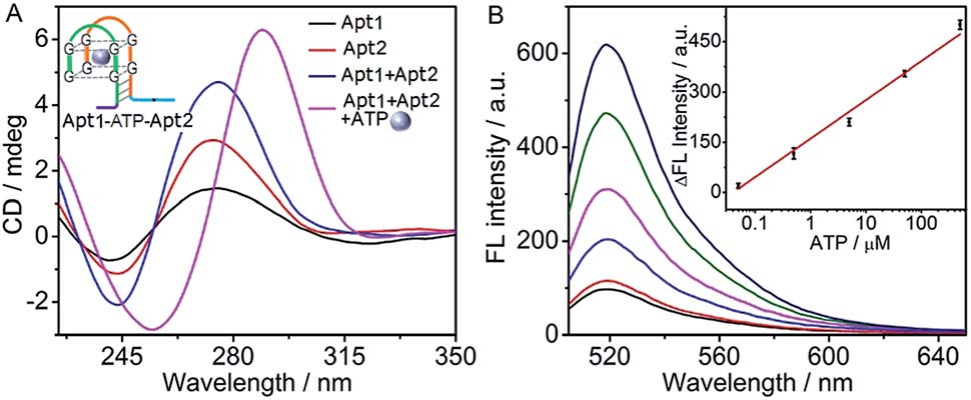
(A) CD spectra of different solutions with the marked components at 5 μM Apt1 and Apt2, and 500 μM ATP. (B) Fluorescence spectra at 0, 0.05, 0.5, 5, 50, 500 μM ATP (from bottom to top). Inset: plot of Δ*F vs.* logarithm of ATP concentration.

Using split fragments of an aptamer as the recognition domains, the cyclic system could sensitively detect an aptamer substrate. Taking ATP as a model, the ATP-binding aptamer was split into two fragments, according to the work of Plaxco's group.^45^ The recognition domains on Apt1 and Apt2 were designed to contain split ATP aptamer subunits which could co-recognize ATP to form a G-quadruplex Apt1–ATP–Apt2 structure^46^ (inset, Fig. 3A). The toehold and branch-migration domains were drawn together in the G-quadruplex structure, which initiated the hairpin assembly and produced a fluorescence signal.

The formation of the G-quadruplex Apt1–ATP–Apt2 structure was verified by circular dichroism (CD) spectroscopy (Fig. 3A). Similar to those of the pure Apt1 and Apt2 strands, the CD spectrum of their mixture showed a positive band near 275 nm and a negative band near 243 nm, which referred to the random coil structure of single-stranded DNA.^47,48^ However, after ATP was added into the mixture of Apt1 and Apt2, the bands of random coil structure disappeared and a dramatically different CD spectrum with the typical bands of the ATP–aptamer G-quadruplex structure (a positive band near 285 nm and a negative band near 250 nm) was observed,^46,49^ indicating the successful formation of the G-quadruplex structure. In addition, the feasibility of the cyclic system for ATP detection was confirmed by fluorescence spectra (Fig. S3 in ESI†), in which the fluorescence signal was only observed in the presence of ATP.

The assay conditions of ATP were optimized to be 6 mM Mg^2+^, 1 μM Q:F, 500 nM H1 and H2, and a reaction time of 180 min (Fig. S4 in ESI†). Here the ATP aptamer interacted with the phosphates of ATP *via* coordination of Mg^2+^, which facilitated the recognition of ATP. However a high concentration would result in a high background signal.^42,50^ Under these conditions, the fluorescence intensity increased with the increasing concentration of ATP (Fig. 3B). The fluorescence intensity was linearly proportional to the logarithm value of ATP concentration over the range of 0.05 to 500 μM with a limit of detection down to 38 nM corresponding to the signal of 3 SD (inset, Fig. 3B). As control, a common experiment without the cyclic assembly of hairpins was designed (Fig. S5A in ESI†). Here, the ATP–aptamer G-quadruplex structure directly dissociated the fluorophore-bearing strand (F′) from the quencher-bearing strand (Q′) *via* the toehold-mediated strand displacement reaction, it showed an ATP detection range from 5 to 1000 μM with a detection limit of 3.6 μM (Fig. S5B in ESI†). Obviously, the proposed cyclic system showed a much lower detection limit (almost 100 times) and 1.4 times higher detection sensitivity according to the slope of the calibration curve in comparison with those without amplification, suggesting the amplification capability of the cyclic system. In addition, the detection limit of ATP with a wide detection range by the cyclic system was comparable to other assays based on G-quadruplex–hemin DNAzyme.^51,52^

The selectivity of the cyclic system towards the detection of ATP was evaluated by comparing the fluorescence intensity of the solutions containing ATP or its analogues such as guanosine triphosphate (GTP), cytidine triphosphate (CTP), and uridine triphosphate (UTP) (Fig. S6 in ESI†). As expected, the cyclic system showed an obvious response to ATP, while a negligible response was observed for all its analogues, indicating the proposed target-driven DNA association for cyclic assembly of hairpins exhibited excellent selectivity for ATP detection.

### AND logic gate

**Fig. 4:**
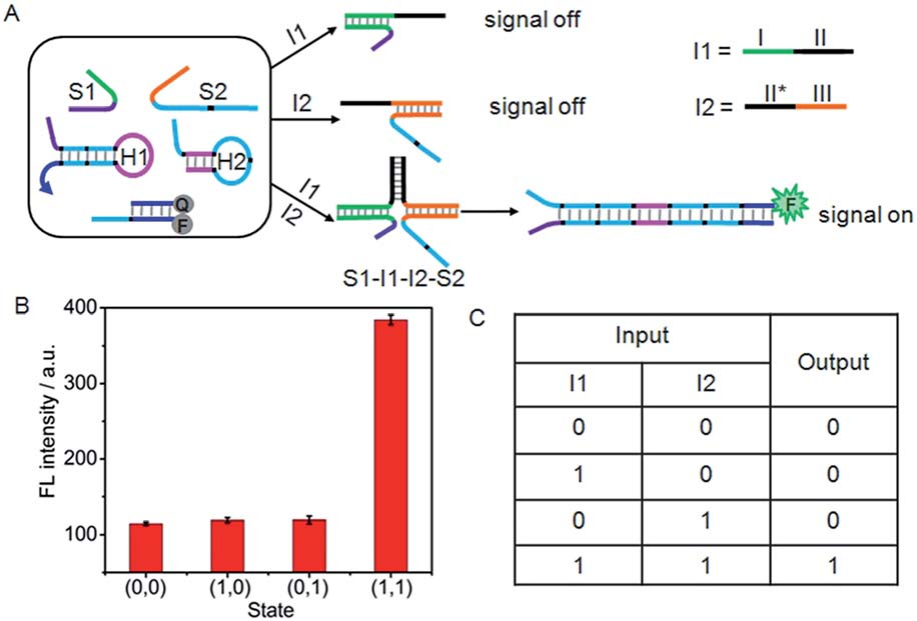
(A) Illustration of the operational “AND” logic gate. (B) Fluorescence intensity measured at 520 nm. (C) Truth table of “AND” logic gate.

Furthermore, a set of logic gates (AND, INHIBIT and NAND) with sensitive operations were constructed based on the cyclic system by designing different input strands to achieve the output signal. The presence and absence of the single-stranded DNA (I1 to I6) were assigned as the inputs of 1 and 0, respectively. As shown in Fig. 4A, an AND gate was demonstrated by employing I1 and I2 as the inputs. I1 was designed to contain two functional domains: the green region I complementary to the recognition domain of S1, and the black region II complementary to the black region II* on I2. In addition, the orange region III of I2 was complementary to the recognition domain of S2. Thus, neither I1 nor I2 alone could co-hybridize with the recognition domains of S1 and S2 and draw the toehold and branch-migration domains into close proximity to initiate the hairpin assembly, leading to a background fluorescence which was defined as “0” output. However, upon the simultaneous treatment of the system with I1 and I2, a DNA four-strand complex (S1–I1–I2–S2) could be formed to bring the toehold and branch-migration domains into close proximity to initiate the hairpin assembly and turn on the fluorescence signal, which produced a high fluorescence intensity defined as “1” output. The output fluorescence signals of the AND gate in the form of a bars representation and the corresponding truth table are shown in Fig. 4B and C, respectively.

### INHIBIT logic gate

**Fig. 5:**
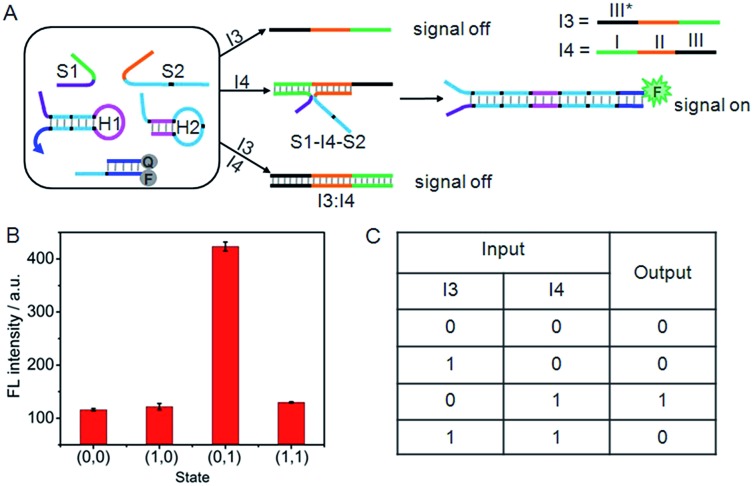
(A) Illustration of the operational “INHIBIT” logic gate. (B) Fluorescence intensity measured at 520 nm. (C) Truth table of “INHIBIT” logic gate.

Similarly, an INHIBIT gate was designed by employing I3 and I4 as the inputs (Fig. 5A). Here, both I3 and I4 were designed to contain three functional domains. The green region I and orange region II of I4 were complementary to the recognition domains of S1 and S2, respectively, and black region III was complementary to the black region III* on I3. The green and orange regions on I3 contained the same base pairs as the recognition domains of S1 and S2. Thus, I4 could not only hybridize with I3 to form an I3:I4 duplex, but also co-hybridize with S1 and S2 to form the DNA three-strand complex (S1–I4–S2). Upon treatment of the system with I3 alone, the “0” output would be observed. In contrast, because I4 triggered the formation of S1–I4–S2 that could drive the DNA association and initiate the hairpin assembly, a high fluorescence signal could be produced. Thus the treatment of the system with I4 alone would lead to the “1” output. However, the ability of I4 to form S1–I4–S2 would be inhibited by the coexistence with I3 due to the formation of the stable I3:I4 duplex, hence, treatment of the system with both I3 and I4 would result in the “0” output. Fluorescence spectra were used to evaluate the operation of the INHIBIT gate (Fig. 5B). As expected, a large fluorescence signal was obtained only when I4 was solely added, which was in accord with proper execution of the INHIBIT gate. The corresponding truth table is shown in Fig. 5C.

### NAND logic gate

**Fig. 6:**
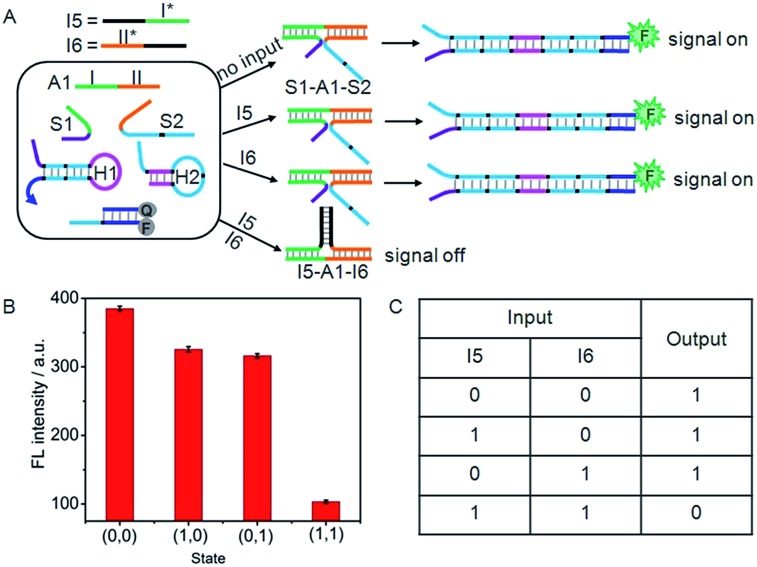
(A) Illustration of the operational “NAND” logic gate. (B) Fluorescence intensity measured at 520 nm. (C) Truth table of “NAND” logic gate.

A NAND gate offers a true “1” output with all combinations of binary inputs except for the (1,1) input state which gives a false “0” output. For this purpose, an auxiliary single-stranded DNA (A1) was employed to construct the NAND gate (Fig. 6A). A1 included two functional domains, the green region I (16 bases) and orange region II (17 bases), which were complementary to the recognition domains of S1 and S2, respectively. Here, I5 and I6 served as the inputs of the NAND gate. The green region I* (14 bases) and orange region II* (15 bases) on I5 and I6 contained two fewer base pairs than the green region I and orange region II on S1 and S2, respectively, and the black regions (12 bases) on I5 and I6 were complementary to each other. Thus, in the absence of any input, the co-added A1 could hybridize with S1 and S2 to form the DNA three-strand complex (S1–A1–S2), while in the presence of both I5 and I6, the hybridization of the inputs with the co-added A1 produced a stable three-strand complex of I5–A1–I6. It was noted that the S1–A1–S2 structure was energetically favoured in the presence of either I5 or I6, since green region I (16 bases) and orange region II (17 bases) on S1 and S2 were two bases longer than the green region I* (14 bases) and orange region II* (15 bases) on I5 and I6. The S1–A1–S2 structure could drive DNA association, and thus initiated the assembly of hairpins.

As a result, this system would yield the true “1” output (a threshold value was set at 150 to judge the positive and negative output signals) in the absence of I5 or/and I6 (Fig. 6B and C). However, due to the formation of the I5–A1–I6 complex, treatment of the system with both I5 and I6 (1,1) would result in the false “0” output.

## Conclusions

The present study designed a target-driven DNA association for initiating the cyclic assembly of hairpins to amplify the detection signal of the target and activate the DNA logic gates. DNA association *via* simultaneous recognition of the target by two ssDNA and the initiated hairpin assembly have been demonstrated. The co-recognition can be achieved in the presence of a target nucleic acid or aptamer substrate by using split fragments of DNA or aptamer as the recognition domains. Benefiting from the cyclic amplification, the proposed system can perform amplified biosensing and logic gate operations without need of enzyme. The cyclic system can conveniently be extended to detect other analytes such as proteins, RNA and metal ions, by modifying the two ssDNA with corresponding recognition domains. The proposed AND, INHIBIT and NAND logic gates show high sensitivity and flexible operations. Therefore, the designed system provides a versatile platform for sensitive detection and logic gate operations, and shows potential applications in bioanalysis and disease diagnosis.

## Experimental section

### Materials and reagents

ATP, GTP, CTP, UTP and all the oligonucleotides were purchased from Shanghai Sangon Biotechnology Co. Ltd. (China). The oligonucleotides were purified using high performance liquid chromatography and their sequences are listed in Table S1 in the ESI.† All other reagents were of analytical grade and used without further purification. Ultrapure water obtained from a Millipore water purification system (≥18 MΩ, Milli-Q, Millipore) was used in all assays. Phosphate buffer saline (PBS, 10 mM pH 7.4) containing 0.1 M NaCl was prepared by mixing the stock solutions of NaH_2_PO_4_ and Na_2_HPO_4_.

### Apparatus

PAGE analysis was performed on an Electrophoresis Analyser (Liuyi Instrument Company, China) and imaged on a Bio-rad ChemDoc XRS (Bio-Rad, USA). CD spectra were conducted on a JASCO J-810-150S circular spectropolarimeter (Tokyo, Japan), of which the lamp was always kept under a stable stream of dry purified nitrogen (99.99%) during experiments. Fluorescence measurements were conducted by scanning from 510 to 650 nm with a step of 1 nm at an excited wavelength of 492 nm on a F900 fluorescence RF-5301 PC spectrofluorophotometer (Shimadzu, Japan).

### PAGE analysis

A 10% native polyacrylamide gel was prepared using 5× TBE buffer. The loading sample was a mixture of 7 μL DNA sample, 1.5 μL 6× loading buffer, and 1.5 μL of UltraPower™ dye. Before injection into the polyacrylamide hydrogel, the loading sample was placed for 3 min. The gel electrophoresis was run at 90 V for 1 h. The resulting board was illuminated with UV light and photographed with a Molecular Imager Gel Doc XR.

### Detection of DNA and ATP

S1, S2, H1, and H2 were annealed by heating at 95 °C for 5 min and then slowly cooled down to room temperature over 3 h. Apt1 and Apt2 were heated to 95 °C for 5 min, cooled on ice quickly and then stored at 4 °C. Q:F duplex was formed by mixing single-stranded Q and F strands followed by heating at 95 °C for 5 min and then slowly cooling down to room temperature over 3 h. For DNA sensing, 100 nM S1 and S2, 200 nM H1 and H2, and 200 nM Q:F duplex were mixed with target DNA at different concentrations in 10 mM PBS and incubated for 1 h at 37 °C. For ATP sensing, 250 nM Apt1 and Apt2, 500 nM H1 and H2, and 1 μM Q:F duplex were mixed with ATP at different concentrations in 10 mM PBS and incubated for 180 min at 37 °C. Afterwards, the fluorescence measurements were performed by scanning from 510 to 650 nm.

### DNA logic gate operations

After 100 nM S1 and S2, 200 nM H1 and H2, and 200 nM Q:F duplex were mixed, different pairs of input DNA at 50 nM were added for 1 h incubation at 37 °C. The DNA logic gates were observed by fluorescence measurements.
