# Supplementary material for: Target-driven DNA association to initiate cyclic assembly of hairpins for biosensing and logic gate operation
Source: Chem Sci. 2015 May 12;6(7):4318–23. doi: 10.1039/c5sc01215e (PMC5707516; doi:10.1039/c5sc01215e)
Supplement: Supplementary file 1 [file SC-006-C5SC01215E-s001.pdf]

Electronic Supplementary Material (ESI) for Chemical Science.
This journal is © The Royal Society of Chemistry 2015

Electronic Supplementary Information for

# Target-driven DNA association to initiate cyclic assembly of hairpins for biosensing and logic gate operation

**Yuehua Guo, Jie Wu, and Huangxian Ju***

*State Key Laboratory of Analytical Chemistry for Life Science, School of Chemistry and Chemical Engineering, Nanjing University, Nanjing 210093, P.R. China. Tel/Fax: +86 25 83593593; E-mail: hxju@nju.edu.cn*

## Contents

1. Supporting table……………………………………..……………………………2

2. Supporting figures……………………………………..……………………………3

## 1. Supporting table

***Table S1*** Sequences of oligonucleotides used in this work.

| **Name** | **Sequence (from 5' to 3')** |
|---|---|
| S1 | GAGACCATCAATGAGGTCACTGAC |
| S2 [a] | CGACATCTAACCTAGC*T*AAGCTGCAGAATGGGAT |
| Target DNA | ATCCCATTCTGCAGCTTCCTCATTGATGGTCTC |
| Apt1 | ACCTGGGGGAGTATGTGAGG*T*TCACTGAC |
| Apt2 | CGACATCTAACCTAGCCCTCACAGCGGAGGAAGGT |
| H1 | GTCAGTGAGCTAGGTTAGATGTCGCCATGTGTAGACGACATCTAACCTA GCCCTTGTCATAGAGCAC |
| H2 | AGATGTCGTCTACACATGGCGACATCTAACCTAGCCCATGTGTAGA |
| Q | CCTTGTCATAGAGCACTCG-Dabcyl |
| F | FAM-CGAGTGCTCTATGACAAGGGCTAGGTT |
| Q' | Dabcyl-CGACATCTAACCTAGC |
| F' | GTCAGTGAGCTAGGTTAGATGTCG-FAM |
| I1 | CACTCGACAGAT*T*CCTCATTGATGGTCTC |
| I2 | ATCCCATTCTGCAGCTTATCTGTCGAGTG |
| I3 | TAGCACCAGTTGAGACCATCAATGAGGAAGCTGCAGAATGGGAT |
| I4 | ATCCCATTCTGCAGCTTCCTCATTGATGGTCTCAACTGGTGCTA |
| I5 | GACCATCAATGAGGTATAATAAATTT |
| I6 | AAATTTATTATAAAGCTGCAGAATGGG |
| A1 | ATCCCATTCTGCAGCTTCCTCATTGATGGTCTC |

[a] The italicized T in the table is applied to increase the stability of DNA duplex.

## 2. Supporting figures

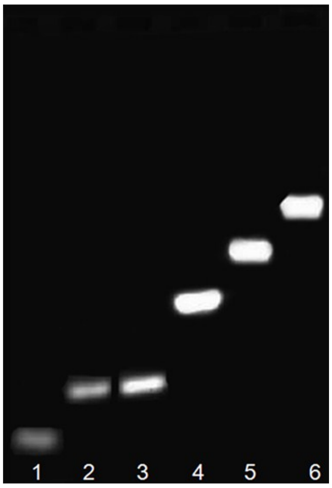


**Fig. S1** Polyacrylamide gel electrophoresis (PAGE) analysis of the formation of DNA three-strand complex. Lanes 1 to 6 correspond to 5.0 μM S1, S2, target DNA, S1+target DNA, S2+target DNA, and S1+S2+target DNA, respectively.

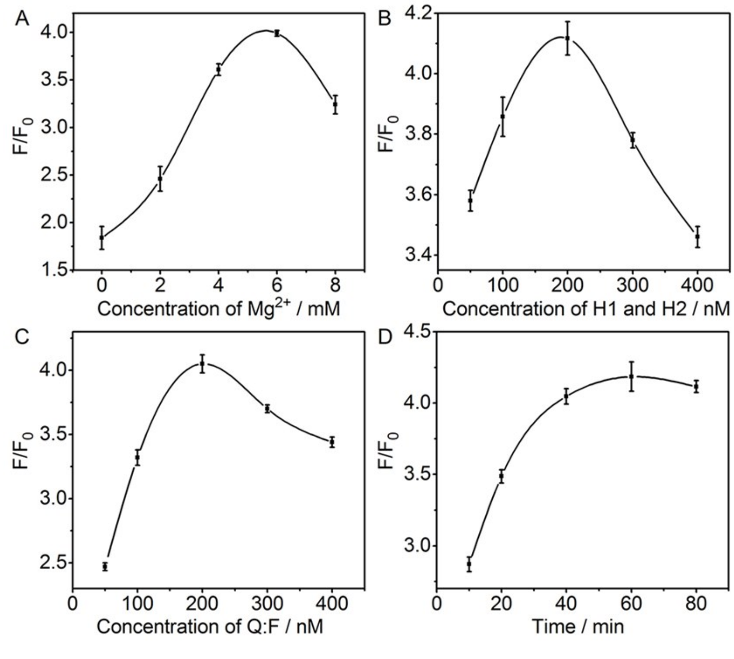


**Fig. S2** Condition optimization for DNA detection: concentrations of (A) $Mg^{2+}$, (B) hairpins and (C) Q:F duplex, and (D) reaction time. Error bars represent the standard deviation of three parallel experiments at 100 nM S1 and S2 and 50 nM target.

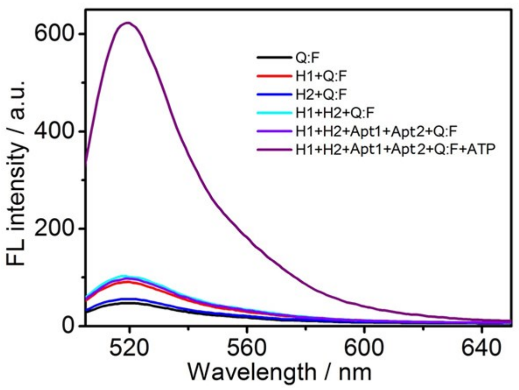


**Fig. S3** Feasibility of ATP detection using the designed system at 500 nM H1 and H2, 250 nM Apt1 and Apt2, 1 μM Q:F and 500 μM ATP.

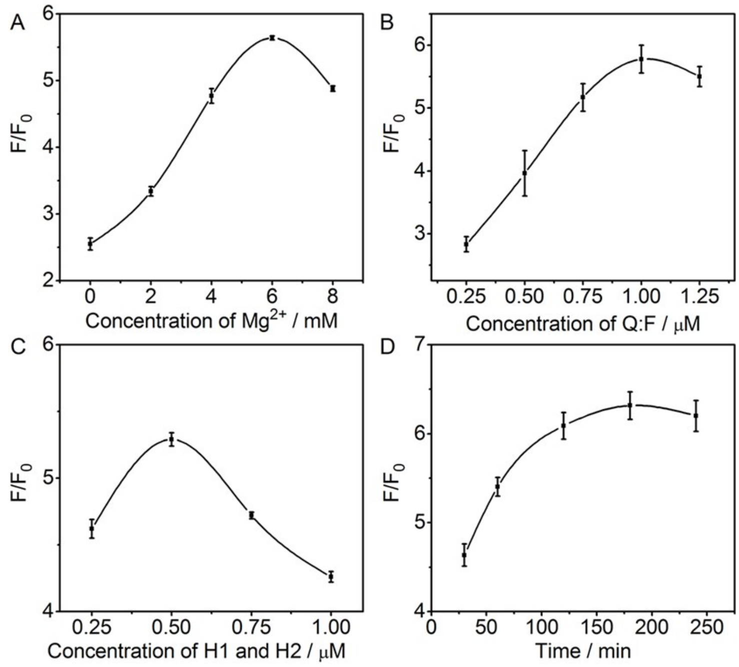


**Fig. S4** Condition optimization for ATP detection: concentrations of (A) $Mg^{2+}$, (B) Q:F duplex and (C) hairpins, and (D) reaction time. Error bars represent the standard deviation of three parallel experiments at 250 nM Apt1 and Apt2 and 500 μM ATP.

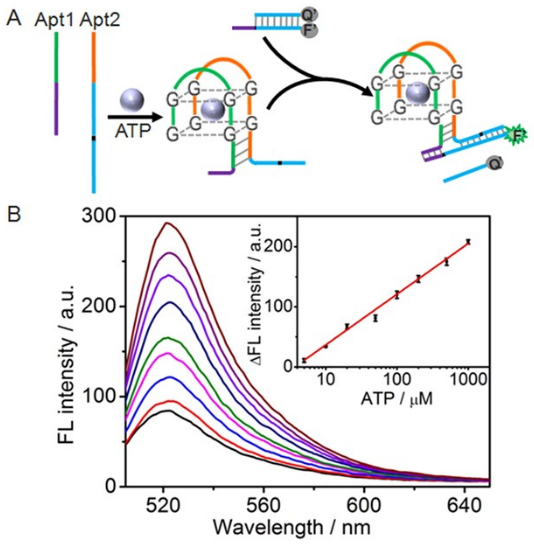


**Fig. S5** Detection of ATP without amplification. (A) Scheme and (B) fluorescence spectra at 0, 5, 10, 20, 50, 100, 200, 500 and 1000 μM ATP (from bottom to top). Inset: plot of $\Delta F$ *vs.* logarithm of ATP concentration ranging from 5 to 1000 μM.

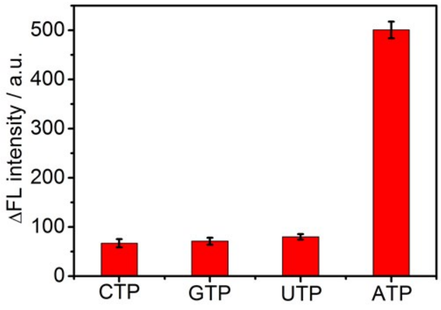


**Fig. S6** Selectivity of ATP analysis. The error bars represent the standard deviation of three measurements.
